# Modeling the Contribution of Multiple Micronutrient Fortification of Salt to Daily Nutrient Intake Among the Ethiopian Population

**DOI:** 10.1016/j.cdnut.2024.103794

**Published:** 2024-06-07

**Authors:** Semira Mitiku Saje, Dawd Gashu, Edward JM Joy, Katherine P Adams, Tibebu Moges, Masresha Tesemma, E Louise Ander

**Affiliations:** 1 Center for Food Science and Nutrition, Addis Ababa University, Addis Ababa, Ethiopia; 2 Faculty of Epidemiology and Population Health, London School of Hygiene and Tropical Medicine, Keppel Street, London, United Kingdom; 3 Institute for Global Nutrition, Department of Nutrition, University of California, Davis, CA, United States; 4 Food Science and Nutrition Research Directorate, Ethiopian Public Health Institute, Gulele Sub City, Addis Ababa, Ethiopia; 5 School of Biosciences, University of Nottingham, Sutton Bonington Campus, Loughborough, Leicestershire, United Kingdom; 6 Inorganic Geochemistry, Center for Environmental Geochemistry, British Geological Survey, Nottingham, United Kingdom

**Keywords:** micronutrients, salt fortification, nutrient modeling, folate, zinc

## Abstract

**Background:**

Salt is an affordable commodity and has wide coverage regardless of economic and social status and, hence, could be suitable vehicle for multiple micronutrient fortification.

**Objectives:**

This study aimed to simulate the contribution folic acid and zinc fortification of iodized salt to nutrient intake among the Ethiopian population.

**Methods:**

The 2013 Ethiopian National Food Consumption Survey and various food composition tables were used to estimate baseline individual-level micronutrient intake. Usual intake was estimated using the Simulating Intake of Micronutrients for Policy Learning and Engagement macro tool. Discretionary salt consumption was calculated from total salt intake estimated using urinary sodium excretion. Fortificant addition rates were set to obtain maximum nutrient intake while simultaneously constraining that population with intake above the tolerable upper intake level to <5%. Addis Ababa and Somali (*N* = 2271), the regions with relatively the lowest and highest micronutrient deficiency prevalence in Ethiopia, were selected.

**Result:**

Baseline median intake of Zn was below the estimated average requirement for all demographic groups. Inadequate Zn intake ranged from 73% to 99%, the highest prevalence being observed among women in lower class of wealth quintiles from Somali region. Dietary folate inadequacy was as low as 2% among men in Addis Ababa but almost all (99%) women from Somali region had inadequate folate intake. Calculated discretionary salt intake was 7.5 g/d for adult men and women and 3.4 g/d for children. With addition 0.8 mg Zn and 30 μg of folic acid per gram of salt, multiple salt fortification is estimated to reduce Zn inadequacy by 38 percentage points in urban areas and19 percentage points in rural areas. Modeled reduction in folate inadequacy were 18% in urban areas and 22% in rural areas.

**Conclusions:**

Multiple salt fortification could be an effective approach to address micronutrient adequacy in Ethiopia given efficacious, technological, and economical feasibility.

## Introduction

Salt is an affordable commodity that is consistently purchased and consumed regardless of economic circumstances and social settings [1,2]. Compared with other fortifiable food vehicles, it has wider coverage and reaches large populations. It often originates from a small number of sources, which can facilitate efficient and effective monitoring and regulation. On the contrary, other commonly fortified food vehicles such as wheat flour often have limited reach to the poorest segment of the society and rural communities where the delivery system of fortified food is absent or not developed [3,4].

Although historically salt has been fortified with iodine alone, it can be fortified with multiple micronutrients such as iron, zinc folic acid, and vitamin B-12 [[5], [6], [7]]. Studies show that micronutrients added to table salt can be stable during cooking and after several months of storage [8,9]. In addition, its effectiveness in enhancing micronutrient intake and alleviation of deficiency has been reported [[10], [11], [12]].

Several studies and reports are available to support effective planning and implementation of multiple micronutrient fortified salt (MFS). Consumer acceptability tests (taste, color, and appearance) have shown that quadruple (iodine + iron + vitamin B-12 + folic acid) and double (iodine + iron) fortified salt (DFS) had comparable score with single fortified salt (iodized salt) [13]. However, it is understandable that color and taste of fortified salt depends on addition level and combination of the fortificants. Findings of a study that systematically evaluated DFS programmatic experiences around the world revealed that color change was of an issue in 9 of 14 programs included in the study. In addition, lack of quality standards and regulatory protocols were among the factors limiting large-scale implementation of DFS [14]. Several efficacy and effectiveness studies reported that consumption of DFS or MFS has increased micronutrient intake, cognitive performance, and hemoglobin concentration and reduced the risk of anemia incidence [[10], [11], [12],[15], [16], [17]].

A technology to simultaneously deliver multiple micronutrients (iron, zinc, iodine, folic acid, and vitamin B-12) through fortification of salt has been developed [18,19]. Few studies reported cost implication of multiple micronutrient fortification of salt (MFS). For example, Modupe and Diosady [19] reported the additional cost due to folic acid, iron, and iodine fortification of salt to be USD 0.27/person per year. The authors also suggested that the cost could be further reduced with large-scale production. The cost benefit ratio for DFS with iron and iodine in India to increasing iron and reducing the risk of anemia incidence was estimated between 2.4:1 and 5:1 [20]. However, it is important to note that cost benefit estimates tend to differ by country and baseline micronutrient status.

In Ethiopia, the prevalence of multiple micronutrient deficiency is high. Based on serum data, 72% of the Ethiopian population have Zn deficiency [21]. Furthermore, 77.9% women had low red blood cells folate concentration; hence, an increased risk of neural tube defect (NTD)-affected pregnancies [22]. A recent systematic review and meta-analysis of the literature showed that in Ethiopia the prevalence of all NTDs was 71 per 10,000 births, which is 2 times greater than the estimated prevalence in East Africa [23]. On the contrary, the recent food and nutrition survey report also shows that only 8% and 9% of reproductive age women in Ethiopia have iron and vitamin B12 deficiency [24].

When Ethiopia introduced mandatory salt iodization during 2011, salt was iodized by spraying dissolved potassium iodate onto unrefined coarse salt at the production salt. This later evolved into the formation of central iodization facilities where small salt producers supply salt into such infrastructures for proper iodization. Today, there are >17 central iodization facilities that are strategically situated in the country considering proximity to origin of salt, market, road, and electricity access [25]. This situation, combined with a fairly easy regulatory environment for salt iodization owing to the majority of table salt consumed by Ethiopian households coming from 1 source (Lake Afdera) has led to high coverage of iodized salt across the country, with recent evidence showing that 89% of households consume iodized salt [25]. However, the recent Ethiopian Food and Nutrition survey report indicated that only half (51%) of the households in the country consume adequately iodized salt (15–40 ppm) and the iodine concentration in table salt samples for a quarter of households were below the minimum acceptable level (15 ppm). In addition, the distribution of households with access to iodized salt is not uniform. Somali region has the highest percentage (86%) of households with inadequately iodized salt (<15 ppm) while only 7% of households in Addis Ababa consume salt with iodine content <15 ppm. Difference in iodized salt coverage was not observed by urbanicity [26]. Furthermore, a survey report also shows that 59% and 41% of salt samples collected from the Ethiopian households and the market, respectively, had greater grain size (coarse salt) [26], which may affect homogenous mixing and added premix stability.

In 2022, Ethiopia introduced mandatory wheat flour fortification, but fortifiable wheat flour is only consumed by 28% of the population, mainly residing in urban areas [27]. In addition, micronutrient supplementation is not common among the majority of the population in Ethiopia. Less than half (44.4%) of young children receive high dose vitamin A and only 41.4% of pregnant women are taking iron–folic acid supplementation [28,29]. In addition, three-quarters of women and over 90% of children do not have the minimum acceptable dietary diversity [30]. As such, expanding Ethiopia’s salt iodization program to fortify salt with additional micronutrients may be one of the most effective available strategies to improve the micronutrient adequacy of diets across large segments of the population. Given the public health significance of zinc and folate deficiencies in Ethiopia, in this study, we present results of modeling analyses to assess the potential impact of expanding Ethiopia’s salt fortification program to include zinc and folic acid. This is because the deficiency of zinc and folic acid is a public health importance among the Ethiopian population and the technological feasibility for addition of these micronutrients to salt exists.

## Methods

The 2013 Ethiopian National Food Consumption Survey (ENFCS) data were used to estimate baseline individual-level dietary intake. Briefly, the ENFCS was a nationally representative cross-sectional survey conducted during 2013 to provide information to the government about food access and utilization. The survey population was drawn from households randomly selected to be representative of all 9 regions (Afar, Amhara, Benshangul-Gumuz, Gambella, Oromia, Somali, Southern Nations Nationalities and People’s, Tigray, and Harari) and 2 city administrations (Addis Ababa and Dire Dawa). The clusters were selected from rural and urban sites to ensure collection of dietary habits from a range as broad as possible of different ethnic, geographic, socioeconomic, and cultural settings.

The sampling included 26 households from each of 324 enumeration areas, totaling 8424 households. The target populations were young children (6–35 mo) and their closest female caregivers (15–49 y) and (in ∼30% of urban households) their closest male caregiver or relative (19–45 y). Detailed methodologic description of the survey can be found in the ENFCS technical report [31]. Briefly, dietary and socioeconomic data were collected during 2013. The dietary data collection included 1 reported individual-level 24-h dietary recall per participant. Specific types and amounts of foods consumed by the participants 24 h before the survey were recorded. The questionnaire was developed based on internationally recognized multiple pass method described by Gibson and Ferguson [32]. Each interview involved a stepwise series of questions, normal household utensils, food substitutes (play dough, flour, lentils, and water) and pictures of most commonly consumed foods in the specific regions to improve the memory of the respondents and assist in completing the questionnaires. A digital food scale was used to measure the amount of food consumed and of ingredients used in food preparation.

Interviews were conducted on all 7 d of the week to capture variation in intakes across various days of the week. Interviews were conducted in the first language of the person being interviewed; enumerators were selected to speak the predominant regional first language, but local translators were used when needed.

In this study, data from ENFCS for Somali region and Addis Ababa city administration (selected because they have the highest and lowest micronutrient deficiency prevalence, respectively) were used to estimate the baseline dietary intake among all demographic groups [*n* = 2271, 836 children (1–3 y), 110 men (19–45 y), and 1325 women of reproductive age (15–49 y, including pregnant and lactating women)] to simulate the potential contribution of MFS to daily iodine, folic acid, and zinc intake and the prevalence of inadequacy of the mentioned nutrients.

A socioeconomic index was constructed using principal components analysis based on household asset data including ownership of a number of consumer items. Socioeconomic status (SES) quintiles were calculated by assigning the household score to each household member and then dividing the ranking into 5 equal categories, each comprising 20% of the population [33]. SES calculation was applied to the national data but the present analysis only considered 2 regions hence, even distribution of wealth index quintiles is not expected.

### Dietary data

Zinc content of the foods and beverages consumed by the participants during the survey was estimated from the Ethiopian food composition table as reported in the consumption survey [31]. In addition, because, the Ethiopian food composition table (FCT) does not include estimates of folate content, folate contents of consumed foods were estimated from FCTs of Malawi [34], Kenya [35], Tanzania [36], and the United Kingdom [37]. Because the ENFCS collected single-day food consumption, it was not possible to directly estimate within- and between-person variation; hence, within-person variation was obtained from nutrient variance database [38]. The selection of studies for variance imputation involved a comparison of various study characteristics such as study site, study year, season of data collection, and income category based on the 2020 World Bank country income classification. Children of both sexes, pregnant women, and residency (urban and rural) were considered in the analysis. Imputed variances for zinc and folic acid were taken from Uganda and Cameroon studies. The variance estimates used in the present analysis were 0.47 and 0.61 for zinc for children and adults, respectively, and 0.72 and 0.89 for folate for children and adults, respectively [39,40].

Usual intake was estimated using the 1-d Simulating Intake of Micronutrients for Policy Learning and Engagement (SIMPLE) macro tool [38]. Subgroup analysis for adults was done using sex, residence, and SES. We also considered the effect of covariates including age, sex, region, SES, sickness, consumption on weekend, unusual consumption, and place of residence. Prevalence of inadequate and excess intake of micronutrients of interest were estimated by comparing against the estimated average requirement (EAR) and the tolerable upper intake level of the micronutrients. EAR and upper intake level values for folate and Zn (assuming low bioavailability) were obtained from Institute of Medicine [41] and European Food Safety Authority [42].

### Salt consumption

Because salt consumption was not collected in the ENFCS, the consumption among adults was estimated using the Ethiopian national WHO STEPS report, based on spot urinary sodium excretion analysis and adjusted for hydration status. The Ethiopian STEPS survey is a cross-sectional and nationally representative study conducted during 2015 by using WHO STEP wise approach surveillance of adults (15–69 y, *n* = 6761) to measure risks to noncommunicable disease [43]. However, the survey reports total salt consumption from various sources (food, water, and table salt). In this study, 90% of the total salt consumption was considered to come from discretionary salt and manufactured food items [44]. Moreover, in line with discretionary salt consumption quantities recommended by the salt reduction initiative, we modeled another scenario in which discretionary salt consumption was assumed to be 5 g/d among adults [45]. Salt consumption for children was calculated in proportion to their energy intake estimated from the food consumption survey data [31]. Recommended salt intake was the same for all adults (i.e., 5 g/d) and almost half of adult’s consumption for children, based on energy intake irrespective of sex and place of residence.

### Fortification level

Level of premix addition was calculated taking into account the factors such as the size of the nutrient gap between nutrient intake from usual diet and EAR of nutrients specified by demographic groups, the amount of daily discretionary salt consumption [45], technological feasibility and cost analysis [19], sensory (e.g., color and taste) attributes of fortified salt and consumer acceptability [8,9,19,[46], [47], [48]]. In addition, fortification levels were set with the intention to maximize nutrient adequacy but the risk of excess intake was limited to <5% of the population [4].

### Ethical approval

The study was approved by the Research Ethical Review Committee at the Ethiopian Public Health Institute (Protocol EPHI-IRB-140–2018) and Institutional Review Board at Addis Ababa University (Reference CNCSDO/192/14/21).

## Results

Data for 2271 participants: 836 children (1–3 y), 110 men (19–45 y); and 1325 women of reproductive age including pregnant and lactating women were analyzed in this study. Among them, 1230 were from Addis Ababa. Participants from Addis Ababa were categorized only in the upper or middle upper economic statuses quintiles. Sociodemographic and economic characteristics of the participants are indicated in Table 1.

**TABLE 1:** Characteristics of study participants from Ethiopian National Food Consumption Survey 2013

	Addis Ababa, *n* (%)	Somali, *n* (%)	Combined, *n* (%)
Demographic group			
Children (1–3 y)	422 (34)	414 (40)	836 (37)
Women (15–49 y)	722 (59)	603 (58)	1325 (58)
Male (19–45 y)	86 (7)	24 (2)	110 (5)
Residence			
Urban	1230 (100)	177 (17)	1407 (62)
Rural	0 (0)	864 (83)	864 (38)
Socioeconomic status			
Highest	1111 (90)	97 (9)	1208 (53)
Upper middle	119 (10)	161 (15)	280 (12)
Middle	0 (0)	286 (28)	286 (13)
Lower middle	0 (0)	271 (26)	271 (12)
Lowest	0 (0)	226 (22)	226 (10)
Physiology of women (*n* = 1325)			
Pregnant	22 (3)	69 (12)	91 (7)
Lactating	233 (32)	310 (51)	543 (41)
Nonpregnant and nonlactating	467 (65)	224 (37)	691 (52)
Women’s occupation (*n* = 1325)			
Self-employed	152 (21)	137 (23)	289 (22)
Employed	69 (10)	5 (1)	74 (6)
Housewife	246 (34)	391 (65)	637 (48)
Unpaid family work	51 (7)	19 (3)	70 (5)
Other	204 (28)	51 (8)	255 (19)
Women’s educational status (*n* = 1325)			
Illiterate	113 (16)	515 (85)	628 (47)
Can read and write	15 (2)	7 (1)	22 (2)
Primary school	233 (32)	48 (8)	281 (21)
Secondary school	269 (37)	17 (3)	286 (22)
Other	92 (13)	16 (3)	108 (8)
Household head (*n* = 1325)			
Male	526 (73)	539 (89)	1065 (80)

### Discretionary salt intake and fortificant addition rate

The calculated discretionary salt intake was different across sex and place of residence (Table 2). Based on the recommended amount of discretionary salt intake (5 g/d) the level of fortification per gram of salt was 0.8 mg Zn and 30 μg of folic acid, and 0.6 mg Zn and 22 μg of folic acid.

**TABLE 2:** Micronutrient composition for salt fortification modeling

Region	Demographic group	Calculated discretionary salt intake (g/d)	Nutrient intake from fortified salt (calculated)		Recommended salt intake (g/d)	Nutrient intake from fortified salt (recommended)	
			Zinc (mg)	Folic acid (μg, DFE)		Zinc (mg)	Folic acid (μg, DFE)
Addis Ababa	Children	3.0	1.8	66	2.3	1.8	69
	Women	6.7	4.0	147	5.0	4.0	150
	Men	8.5	5.0	187	5.0	4.0	150
Somali	Children	2.4	1.4	53	2.3	1.8	69
	Women	5.3	3.2	117	5.0	4.0	150
	Men	6.8	4.0	150	5.0	4.0	150

EAR—zinc: 3.6 mg/d for 1–3 y, 12.7 mg/d for adult men, 9.9–13.7 mg/d for WRA [39]; folate: 120 μg DFE/d for 1–3 y, 320 μg DFE/d for adult men and 320–520 μg DFE/d for WRA [38].

Abbreviations: EAR, estimated average requirement; DFE, dietary folate equivalent; WRA, women of reproductive age.

### Simulated changes to nutrient intake and prevalence of inadequacy

Baseline intake and simulated changes to nutrient intake and prevalence of inadequacy among populations in Ethiopia of different demographic groups is indicated in Table 3. Median intake of Zn was below the EAR for all demographic groups. Prevalence of inadequate Zn intake ranged from 74% (men from Addis Ababa) to 98%, the highest prevalence being observed among women from Somali region. Relatively higher baseline intake was observed among men from Addis Ababa (i.e., 9.3 mg/d). Folate inadequacy was as low as 2% among men in Addis Ababa, while none of the women in the lower socioeconomic status had folate above the EAR.

**TABLE 3:** Simulated changes in nutrient intake and prevalence of inadequate intake due to multiple fortification of salt among the Ethiopian population

	Addis Ababa									Somali								
	Children			Women			Men			Children			Women			Men		
	Baseline	After^1^	After^2^	Baseline	After^1^	After^2^	Baseline	After^1^	After^2^	Baseline	After^1^	After^2^	Baseline	After^1^	After^2^	Baseline	After^1^	After^2^
Median intake																		
Zinc (mg)	2.5	4.3	4.3	6.9	11.1	11.1	9.2	13.2	14.4	1.8	3.7	3.3	4.2	8.3	7.4	5.4	9.3	9.3
Folate (μg, DFE)	126	195	193	457	605	604	552	703	740	62	133	115	159	312	273	207	356	350
Inadequate intake (%)																		
Zinc	77	26	27	85	51	51	74	45	35	89	45	61	98	86	92	93	79	80
Folate	45	3	3	23	3	3	2	0	0	91	35	56	99	84	93	85	37	39

Abbreviations: DFE, dietary folate equivalent.

1 Calculation is based on recommended salt intake.

2 Calculation is based on calculated daily discretionary salt intake.

Multiple fortification of salt can reduce the existing inadequacy intake prevalence by 6–51 percentage points for Zn and 2–56 percentage point for folate. Region wise, regardless of demographic group, salt fortification was predicted to reduce the prevalence of Zn and folate inadequacy, respectively, by 6–44 percentage points and 6–56 percentage points in the Somali region and by 29–51 percentage points, and 2–42 percentage points among those from Addis Ababa. The prevalence of excess intake due to MFS is indicated in Supplemental Table 1.

Among urban adults, men had higher baseline dietary intake of Zn and folate than women participants (Table 4). Salt fortification was estimated to reduce Zn inadequacy by upto 40 percentage points in men and 38 percentage points among women. Reductions in folate inadequacy among urban adults were estimated to be 19 percentage points among women and 9 percentage points among men (Table 4).

**TABLE 4:** Simulated changes to nutrient intake and prevalence of inadequate Zn and folic acid intake by sex among urban adults in response to zinc and folate salt fortification

Micronutrient	Men (*n* = 110)			Women (*n* = 817)		
	Baseline	After^1^	After^2^	Baseline	After^1^	After^2^
Median intake						
Zinc (mg)	8.2	12.3	13.3	6.9	11	11.4
Folate (μg, DFE)	503	650	684	455	604	619
Inadequate intake (%)						
Zinc (mg)	83	54	43	85	53	47
Folate (μg, DFE)	13	5	4	26	7	7

Abbreviations: DFE, dietary folate equivalent.

1 Calculation is based on recommended salt intake.

2 Calculation is based on calculated daily discretionary salt intake.

Baseline dietary Zn and folate intake among urban participants was higher than those residing in rural areas (Table 5). In addition, MFS was predicted to reduce Zn inadequacy by 38 percentage points in urban areas while the reduction in inadequacy was ∼half of that (19 percentage points) in rural areas. On the contrary, folate acid fortification of salt is estimated to reduce folate intake inadequacy by 18 percentage points among population in urban areas and 22 percentage points in rural areas.

**TABLE 5:** Simulated changes to nutrient intake and prevalence of inadequacy of zinc and folate intake by residency among adults in response to multiple salt fortification

	Residence						
	Urban (*n* = 927)			Rural (*n* = 508)			
	Baseline	After^1^	After^2^	Baseline	After^1^	After^2^	
Median intake							
Zinc (mg)	7	11	11.6	5	8.8	9.3	
Folate (μg, DFE)	457	605	622	168	318	334	
Inadequate intake (%)							
Zinc (mg)	85	53	47	97	84	78	
Folate (μg, DFE)	25	7	7	99	81	77	

Abbreviations: DFE, dietary folate equivalent.

1 Calculation is based on recommended salt intake.

2 Calculation is based on calculated daily discretionary salt intake.

Adults in the highest quintile of SES had higher baseline intake of Zn and folate. In addition, Zn and folate fortification of salt resulted in a larger percentage point reduction in inadequacy prevalence among adults in the highest and upper middle SES quintiles than those in the lower SES quintiles (Table 6).

**TABLE 6:** Simulated changes to nutrient intake and prevalence of inadequate zinc and folate intake by quintiles of socioeconomic status among adults in response of multiple salt fortification

	Socioeconomic status														
	Highest (*n* = 792)			Upper middle (*n* = 175)			Middle (*n* = 166)			Lower middle (*n* = 163)			Lowest (*n* = 140)		
	Baseline	After^1^	After^2^	Baseline	After^1^	After^2^	Baseline	After^1^	After^2^	Baseline	After^1^	After^2^	Baseline	After^1^	After^2^
Median intake															
Zinc (mg)	7.3	11.3	11.9	5.7	9.7	10.2	3.9	8	8.5	4	8.1	8.6	5	9.1	9.6
Folate (μg, DFE)	469	618	635	350	497	512	137	287	302	152	300	315	166	320	336
Inadequate intake (%)															
Zinc (mg)	84	50	43	94	74	69	99	90	87	99	90	87	96	80	75
Folate (μg, DFE)	19	3	2	62	28	25	100	90	86	100	86	82	100	83	79

Abbreviations: DFE, dietary folate equivalent.

1 Calculation is based on recommended salt intake.

2 Calculation is based on calculated daily discretionary salt intake.

## Discussion

Deficiencies in multiple micronutrients are very common in resource poor countries. In Ethiopia, 72% of the population are Zn deficient [21] and over two-thirds of women (77.9%) have low red blood cell (RBC) folate concentrations with increased risk of NTD-affected pregnancies [22]. Low intake of micronutrient dense foods is among the common risk factors for micronutrient deficiency [49]. There are few nutrition interventions in place in Ethiopia to effectively tackle micronutrient deficiency. Iron–folic acid supplements are provided to pregnant women for 90+ days as part of antenatal care. In addition to several other micronutrients, Zn and folic acid are included in the Ethiopian mandatory wheat flour fortification program. However, it is estimated that only 28% of the population have access to micronutrient fortified wheat flour [27]. In addition, a recent report showed that only 17% of pregnant women took iron–folic acid supplement during pregnancy [24].

Fortification of salt with multiple micronutrients is getting attention recently among researchers and program implementers because salt is consumed by almost everybody regardless of socioeconomic condition [2]. This modeling article aimed to estimate the potential contribution of MFS among populations from selected regions in Ethiopia with highest and lowest nutrient intake inadequacy.

Data on the amount of discretionary (cooking and table salt) salt intake is important to determine safe and effective addition rates of the specific fortificants. The mean total salt intake among the Ethiopian adult population is estimated at 8.3 g/d [43]. The estimate, however, is based on analysis of sodium excretion using spot urine samples, which is unable to differentiate the source of the sodium. An earlier study estimated that ∼90% of sodium in urine is estimated to be derived from discretionary salt (salt added at the table and during cooking and in processed foods), while the rest is from the natural salt content of foods or salt in drinking water [44]. In this study, the mean discretionary salt intake among Ethiopian adult population was calculated to be 7.5 g/d with higher intake among men than women (8.5 compared with 6.7 g in Addis Ababa and 6.8 compared with 5.3 g in Somali Region). On the contrary, WHO recommends limiting sodium intake to <2000 mg/d equivalent to <5 g/d of salt among adults to reduce the risk of chronic noncommunicable diseases, and salt intake among children be adjusted based on their energy intake relative to adults [45]. Because we selected different fortification levels, the modeled contribution of MFS to micronutrient adequacy was, generally, similar based on calculated salt intake and recommended salt intake.

The level of fortificant addition (22 μg folic acid and 0.6 mg Zn per gram of salt based on calculated discretionary salt intake) considered in this study can supply 35%–58% of the EAR of adult women, men, and children for folate and 53%–58% for Zn and may reduce the prevalence of inadequate intake by 6–51 percentage points for Zn and 2–56 percentage points for folate. These findings are comparable to a simulation study of MFS to usual diets among nonpregnant women in India in which baseline inadequate intakes of iron (46%), zinc (95%), vitamin B-12 (83%), and folate (36%) were reduced by 29, 76, 81, and 36 percentage points, respectively [50]. In the present study, the decision on nutrient addition rate was constrained by factors such as technological feasibility, sensory attribute, and limit excess intake to <5% of population. However, other ongoing or planned dietary–based nutrition interventions were not considered; hence, the addition rate should be interpreted with caution.

Addition rate of micronutrients in other studies ranged from 5 to 52 μg and 1.4 mg/g of salt of folic acid and zinc, respectively [8,9,19,[46], [47], [48]]. Iodine and folic acid can be added through spray solution without the need for changing the existing fortification equipment [48]. However, color change was observed on the fortified salt when folic acid is added as spray at a rate of 12.5–25 μg/g of salt [8], but the same amount did not show the color change when added as premix [48]. To our knowledge, studies focusing on the technology of triple fortification of salt with iodine, folic acid, and zinc are not available. However, a study attempting to simultaneously fortify salt with iodine, iron, and zinc reported that iodine was lost when zinc was added in the spray solution but researchers observed a viable outcome due to the encapsulation of zinc and iron [51]. Another study at the University of Toronto successfully developed quintuple fortified salt containing folic acid, vitamin B-12, iron, and zinc using microencapsulation technology on iodized salt [52]. This may cause potential difference in the feasibility and cost-effectiveness of DFS with iodine and folate compared with fortification with the inclusion of iodine, folic acid, and zinc.

Ideally, nutrition interventions such as large-scale food fortification should be equitable and provide all segments of the population the ability to consume nutritious, affordable, and culturally acceptable diet. In this study, MFS is predicted to uplift 38% of the population in urban areas compared with 19% in rural areas from Zn inadequacy to adequate intake. But the effect of folic acid fortification to increasing nutrient adequacy in urban and rural areas was comparable (18% compared with 22%). The mode level of fortification was sufficient to reduce the prevalence of inadequate folic acid intakes among children, women, and men in Addis Ababa to <5%. In Somali region, however, owing to lower baseline intakes, while inadequate intakes of folic acid reduced substantially, they will be remained prevalent among children (35%), women (84%), and men (37%), and it is likely that these groups would benefit from a higher level of fortification. From an equity perspective, a higher level of folic acid fortification maybe warranted to deliver a greater reduction in the risk of folate deficiency among middle, lower middle, and low SES households. The difference in modeled reduction in the prevalence of inadequate intakes results from differing baseline nutrient intakes from usual diets, and differing levels of discretionary salt intake. Salt is an inexpensive commodity that is consumed by almost all individuals across sex, residency, and socioeconomic status. However, the incremental cost of salt fortification with multiple micronutrients in the Ethiopian context is not known, and additional research to estimate this cost should be conducted to ensure costs are affordable to all stakeholders, including consumers in low-income households. In general, MFS could increase intake of limited nutrients and reduce deficiency risks.

This study did not take into account on going and planned nutrition interventions such as large-scale food fortification while setting fortificant addition rates. The estimate for the portion of discretionary salt consumption was obtained from a European country, which their dietary habit is different from the present study participants. In addition, we also reported that salt supply from water was negligible. This might not be the case as Ethiopia has different geology and water chemistry to Europe, and different infrastructure for accessing drinking water. Furthermore, this analysis considered data only from 2 regions and findings are therefore not representative of the nation, and there might be different equity insights if the analysis was done with all regions and administrative cities in Ethiopia. The use of FCT from other countries for nutrient content conversion in this analysis may affects the accuracy of the data. The salt estimate is based on sodium concentration in spot urine samples, which does not catch diurnal variation [53]. This study is based on analysis of individual-level dietary intake assessment of ENFCS that was collected using single 24-h call method, which is unable to account for day to day and seasonal variation [54,55].

## Acknowledgments

The authors are grateful to the Ethiopia Public Health Institute (EPHI) for sharing the Ethiopian National Food Consumption Survey data.

## Author contributions

The authors’ responsibilities were as follows – SMS, DG, EJMJ, KPA, ELA: designed the research; SMS, DG, ELA: conducted the research; TM: supervised dietary consumption data collection; SMS: analyzed the data; SMS, DG: wrote the article and had primary responsibility for final content; and all authors: have read and approved the final manuscript.

## Conflict of interest

The authors report no conflicts of interest. The content is solely the responsibility of the authors and does not necessarily represent the official positions of the Bill & Melinda Gates Foundation.

## Funding

This work was funded by the Bill & Melinda Gates Foundation (INV-002855) through the Micronutrient Action Policy Support (MAPS) Tool Development project. Under the grant conditions of the Foundation, a Creative Commons Attribution 4.0 Generic License has already been assigned. The funder had no role in the design, execution, analyses or interpretation of the data.

## Data availability

Data are available on request from the corresponding author.

## Appendix A Supplementary data

Multimedia component1 Multimedia component1
