# Supplementary material for: Modeling the Contribution of Multiple Micronutrient Fortification of Salt to Daily Nutrient Intake Among the Ethiopian Population
Source: Curr Dev Nutr. 2024 Jun 7;8(7):103794. doi: 10.1016/j.cdnut.2024.103794 (PMC11262160; doi:10.1016/j.cdnut.2024.103794)
Supplement: Multimedia component1 [file mmc1.docx]

Semira Mitiku Saje et al. Modeling the contribution of multiple micronutrient fortification of salt to daily nutrient intake among the Ethiopian population

**Supplementary Table 1**: Simulated changes in prevalence of excess zinc and folic acid intake due to multiple micronutrient fortification of salt among the Ethiopian population

|  |  | zinc |  |  | folate |  |  |
| --- | --- | --- | --- | --- | --- | --- | --- |
|  |  | Baseline | After^†^ | After^‡^ | Baseline | After^†^ | After^‡^ |
| Children | Addis Ababa | 1.1 | 4.7 | 4.9 | 0.7 | 4.4 | 4.6 |
|  | Somali | 0.3 | 1.8 | 0.7 | 0.0 | 0.1 | 0.0 |
| Men | Addis Ababa | 1.3 | 3.5 | 4.4 | 0.4 | 3.2 | 4.7 |
|  | Somali | 0.2 | 0.5 | 0.4 | 0.0 | 0.0 | 0.0 |
| Women | Addis Ababa | 0.1 | 0.2 | 0.2 | 0.0 | 0.4 | 0.5 |
|  | Somali | 0.0 | 0.0 | 0.0 | 0.0 | 0.0 | 0.0 |

^†^Amount is based on recommended salt intake; ^‡^Amount is based on calculated discretionary salt intake
